# Royal Society Congress

**Published:** 1923-06

**Authors:** 


### ROYAL SOCIETY CONVERSAZIONE.

THE first of the two annual conversaziones which are given by the Royal Society in its rooms at Burlington House, took place on Wednesday evening, May 16. There was a large attendance, and throughout the evening interested groups were gathered round the exhibits which were shown in the Officers' Room, the Council Room, the Old Council Room, the Principal Library, the Committee Room, and the Secretaries' Room.

### The Stream-Line Filter.

The exhibit which attracted the most attention was the model of Dr. Hele-Shaw's stream-line filter. The demonstrations proved that this filter, which, as its name indicates, causes the fluid to be filtered to flow with a stream-like motion, can achieve remarkable results in the filtration of oils, and can even remove dyes from their solutions. This is done by forcing the fluid through a pack of sheets closely pressed together, made of material impervious to the fluid itself. The use of sheets of a suitable kind of paper gives a simple and inexpensive filter for laboratory and other work, the filtrate being drawn from one tap and the residuum from another. The filter has the further advantage that it can be washed out without being opened. Professor Vernon Boys's demonstrations of his "Integrating and Recording Gas Calorimeter" proved its value to all consumers of gas, as it keeps a continuous check on the quality of gas supplied by gas companies under the therm system.

### The Diet of a Crocodile.

Among the biological exhibits, the Natural History Museum sent a collection of articles found in the stomach of a crocodile which had been caught in the Duma River, Tanganyika Territory. This odd assortment included metal bracelets, a bead necklace and human bones. Mr. H. Graham-Cannon showed three specimens of water-beetle from which the heads had been cut, and the heads of another kind of water-beetle grafted on. One particularly interesting exhibit in this section was by Mr. H. J. Buchanan-Wollaston, who showed that the markings on herring scales are indications of the age and rate of growth of the fish.

### Five Thousand Photographs a Second.

Two demonstrations were given in the Meeting Room during the evening. The first was by Sir Richard Paget, who showed a set of plasticine models, each of which reproduced one of the English vowel sounds. Professor Wheatstone's talking machine (1834-1837), from the Wheatstone collection at King's College, was shown in operation during this demonstration. The second demonstration showed the amazing possibilities of the cinema. Mr. Walter Heape exhibited photographs taken at the rate of 2,500 and 3,500 a second by a machine designed to take photographs at 5,000 a second. One set of these photographs showed what happens when an electric light bulb is smashed by a hammer—an action that takes not more than one-twentieth part of a second. The impact of the hammer and the flying of the glass into thousands of fragments were as clear and definite as the ordinary film photograph.

